# An Intriguing Correlation Based on the Superimposition of Residue Pairs with Inhibitors that Target Protein-Protein Interfaces

**DOI:** 10.1038/srep18543

**Published:** 2016-01-04

**Authors:** Masakazu Nakadai, Shuta Tomida, Kazuhisa Sekimizu

**Affiliations:** 1 Genome Pharmaceutical Institute Co., Ltd., 1-27-8-1207 Hongo, Bunkyo-ku, Tokyo 113-0033, Japan; 2 Graduate School of Medicine, Dentistry and Pharmaceutical Sciences, Okayama University, 2-5-1 Shikata-cho, Kita-ku, Okayama 700-8558, Japan; 3 Laboratory of Microbiology, Graduate School of Pharmaceutical Sciences, The University of Tokyo, 7-3-1 Hongo, Bunkyo-ku, Tokyo 111-0033, Japan

## Abstract

Druggable sites on protein-protein interfaces are difficult to predict. To survey inhibitor-binding sites onto which residues are superimposed at protein-protein interfaces, we analyzed publicly available information for 39 inhibitors that target the protein-protein interfaces of 8 drug targets. By focusing on the differences between residues that were superimposed with inhibitors and non-superimposed residues, we observed clear differences in the distances and changes in the solvent-accessible surface areas (∆SASA). Based on the observation that two or more residues were superimposed onto inhibitors in 37 (95%) of 39 protein-inhibitor complexes, we focused on the two-residue relationships. Application of a cross-validation procedure confirmed a linear negative correlation between the absolute value of the dihedral angle and the sum of the ∆SASAs of the residues. Finally, we applied the regression equation of this correlation to four inhibitors that bind to new sites not bound by the 39 inhibitors as well as additional inhibitors of different targets. Our results shed light on the two-residue correlation between the absolute value of the dihedral angle and the sum of the ∆SASA, which may be a useful relationship for identifying the key two-residues as potential targets of protein-protein interfaces.

Protein–protein interactions (PPIs) are fundamental to most of the biologic processes involved in health and disease. Thus, a better understanding of PPIs will lead to many practical applications, including the rational design of new therapeutic drugs^1^,^2^,^3^,^4^,^5^,^6^,^7^. Several studies evaluating many aspects of inhibitors targeting PPIs, such as their physicochemical properties^8^,^9^,^10^,^11^,^12^ and their 3D topologies^13^,^14^, have provided useful information. Efficient identification of druggable sites on a target protein at the protein-protein interface, however, remains difficult. Nevertheless, the number of successful small molecule inhibitors has recently increased and many compounds are currently undergoing clinical trials^6^,^7^. Interestingly, situations in which the small molecule mimics one of the protein partners are commonly observed^15^, suggesting that mimicking the orientation of side chains along an α-helix could be useful^15^,^16^,^17^. A recent study also demonstrated that the entry angle into a small pocket at the interface is often quite variable^18^,^19^. Thus, not only the spatial relation between pharmacophores, but also the entry angles of the chains, appear to be important.

Over the past decade, genetic and computational approaches revealed that a hot spot – a residue essential for molecular recognition – plays an important role in PPIs, i.e., its removal impairs or severely compromises binding. The side chains and/or residues at the hot spot deeply protrude into defined small pockets on the partner protein^8^,^20^,^21^,^22^,^23^. Bogan and Thorn reported that hot spots are usually surrounded by a hydrophobic ring known as the *O*-ring^24^,^25^, indicating that the important residues in hot spots utilize hydrophobic interactions to recognize a partner protein. Gonçalves-Almeida *et al.* also suggested that hydrophobic patches in the interface are relevant and important for molecular recognition^26^. Rajamani *et al.* focused on the change in solvent-accessible surface areas (∆SASA) after binding of a side chain of residues to define hot spot residues as those that bury the largest amount of SASAs upon binding, and pointed out that anchor residues provide most of the specificity required for protein-protein recognition^27^.

In this article, we studied a method for identifying the key two-residues (residue pairs) to rationally design inhibitors that target protein-protein interfaces. Our analysis was based on the differences between residues that were superimposed onto small molecule inhibitors (SIRs) and non-superimposed residues (non-SIRs). Publicly available information for 8 drug targets, which included 39 inhibitors that target the protein-protein interfaces of those drug targets and 64 hot spot residues on the interfaces, was obtained. To determine the entry angles of the residues into small pockets on the interfaces and the spatial relationships between the pharmacophores of the PPIs, we focused on two-residue relationships and the dihedral angle (DA) and measured the distances for every two-residue combination. We evaluated shape-related descriptors (i.e., distance, DA) and binding-related descriptors (i.e., hydrophobic interaction, ∆SASA, binding free energy [∆G]) of the residues that were like anchor residues that provided clues for identifying key residue pairs superimposed with the inhibitors targeting the protein-protein interfaces. Finally, we applied the regression equation of this correlation to 4 inhibitors that bind to new sites not bound by the 39 inhibitors as well as additional inhibitors of different targets. Our results shed light on the two-residue correlation between the absolute value of the DA and the sum of the ∆SASAs, which may be a useful signature for identifying key residue pairs as potential targets of protein-protein interfaces. In this report, the protein to which small molecules bind is referred to as the “target protein”, whereas the protein that interacts with the target protein is referred to as the “partner protein”.

## Results

### Basic data: 8 target-partner protein combinations, 39 inhibitors, and 64 residues

**Figure 1:**
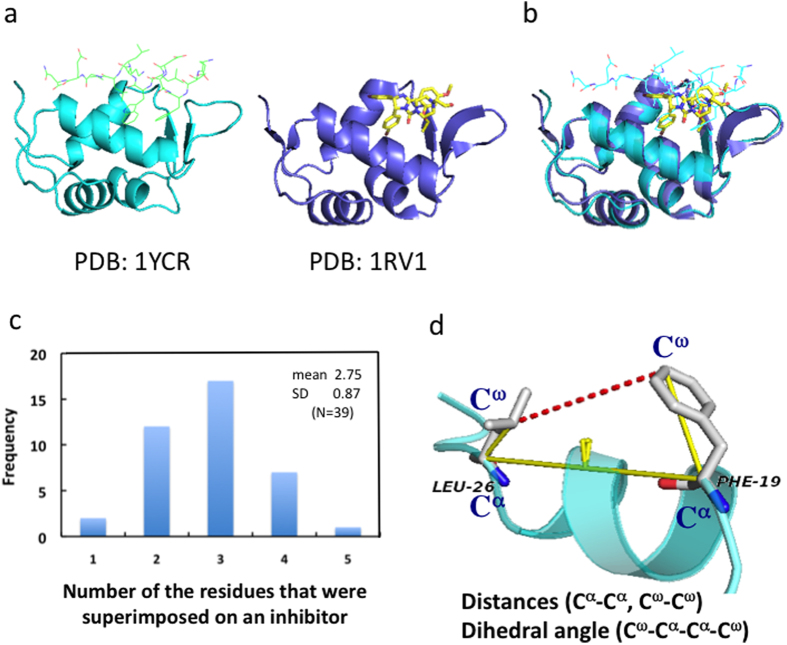
(**a**) Example of a protein-protein structure (pdb:1YCR) and protein-inhibitor structure (pdb:1RV1). (**b**) Example of computational alignment. The alignment between Mdm of the protein–protein complex (pdb:1YCR, green) and Mdm of the inhibitor–protein complex (pdb: 1RV1, purple) is shown. (**c**) The numerical distribution of residues that were superimposed onto an inhibitor. (**d**) Example of a method of measuring the structural data of the residue pairs. The distances of C^α^ – C^α^ and C^ω^ – C^ω^ were measured. The C^ω^ – C^α^ – C^α^ – C^ω^ dihedral angles of the residues are shown (pdb:1YCR). The structural figure was generated using PyMOL (http://www.pymol.org).

To extract solid structural information regarding the target-partner protein combinations from the Protein Data Bank (PDB) database, we used the following four criteria: 1) target proteins for which inhibitor-protein complexes were reported after 2005^28^; 2) basic data of inhibitor-protein complexes and corresponding protein-protein complexes were available from the PDB; 3) inhibitors (small compounds) directly bound to the interface of the target protein; and 4) at least two different crystal structures of the inhibitor-protein complexes were available as of March 31, 2015. Eight target-partner protein combinations were selected according to these criteria and used for further analysis. This information enabled us to compare the properties of the residues of the protein-protein complexes, such as descriptors of their shapes and binding-related parameters, with those of the protein-small molecule (inhibitor) complexes (Table 1). In addition, 39 protein-inhibitor complexes, in which most of the inhibitors were bound to the target proteins in different positions (as shown in Table 1 as PDB IDs of protein-inhibitor complexes), were preferentially selected to avoid structural redundancy between the protein-inhibitor complexes, although this selection method limited the number of protein-inhibitor complexes available for analysis. Sixty-four residues of the eight partner proteins with a ∆SASA (the change in solvent accessible area for each side-chain upon binding^27^) greater than 5 Å^2^ and a predicted –∆G*i* value (estimated free-energy-based scoring function^27^) greater than 1 kcal/mol were selected for further analysis from the ANCHOR database (Table 1, Supplementary Table 1)^27^,^29^,^30^. These residues and inhibitors were on the same interface of the corresponding target proteins. We performed structural alignments between the structures of the target protein in the native protein-protein complexes and the structures of the corresponding protein-inhibitor complexes (Fig. 1a,b). Of the 64 residues, 26 were classified as SIRs based on the thresholds described in the Methods (Supplementary Fig. 1), whereas the remaining residues were classified as non-SIRs. When the secondary structures of the 64 residues were analyzed, 34 residues (53%) belonged to α-helices. This finding is consistent with a previous report showing that most interfaces of the reported PPIs for inhibitor-protein complexes are α-helices (Table 2)^15^,^16^,^17^. The difference in ∆G*i* between the SIRs and non-SIRs was 0.1 kcal/mol, whereas the difference in the hydrophobic effect (HE) of each residue^30^ between the SIRs and non-SIRs was 0.5 kcal/mol. The mean ΔSASA of the SIRs (85.4 Å^2^) was significantly larger than the mean ΔSASA of the non-SIRs (61.9 Å^2^; p = 0.00624; t-test). It seems reasonable that the key descriptors of SIRs are similar to those of the anchor residues *in vivo* because ΔSASA is important to both SIRs and anchor residues^27^.

### Correlations between DA and ∑∆SASA of the superimposed residue pairs

Based on reports about fragment based drug discovery and ligand efficiency, it is assumed that a hit compound with a binding free energy value of –6.9 kcal/mol is generally effective at a concentration of 10 μM^31^,^32^,^33^,^34^. The mean ∆G*i* of the 64 single residues was only –3.5 kcal/mol, however, which does not seem to be enough energy to obtain high-throughput screening hit compounds. We calculated that 2 or more residues were superimposed onto 37 inhibitors (95%), based on the 39 protein-inhibitor complexes (Fig. 1c). This finding led us to hypothesize that correlations exist between two or more residues that might be informative in PPI research. Therefore, we then analyzed the two residues on the same interface of the drug target (i.e., residue pair). To provide a method for measuring the spatial position of a first residue relative to a second residue, we measured three structural parameters, i) distances between C^α^ (alpha carbon atoms of an amino acid) of the first residue and C^α^ of the second residue (C^α^ – C^α^), ii) distances between the C^ω^ (basically, the farthest carbon atom from the C^α^ carbon of an amino acid) of the first residue and the C^ω^ of the second residue pair (C^ω^ – C^ω^), and iii) the DA of C^ω^ – C^α^ – C^α^ – C^ω^ for each residue pair (Fig. 1d). We then classified each residue pair into residue pairs that were both SIRs (superimposed residue pair, SIRP), residue pairs with both non-SIR (non–superimposed residue pair, nonSIR-nonSIR), and residue pairs in which one was superimposed with an inhibitor and the other was not (SIR-nonSIR). 26 SIRs and 38 non-SIRs on 8 target proteins (Table 1) resulted in 35 SIRPs, 90 nonSIR-nonSIRs and 116 SIR-nonSIRs (Supplementary Table 2, note in Supplementary information). To evaluate the effects of binding-related parameters, we calculated the sum of HE (∑HE), ∆G*i* (Σ∆G*i*), and ∆SASA (∑∆SASA) for each residue pair. The mean distances between the atoms (C^ω^ – C^ω^, C^α^ – C^α^) of the SIRPs were shorter than those of the nonSIR-nonSIR (4.4 Å for C^ω^ – C^ω^, p = 0.00039, t-test; 3.7 Å for C^α^ – C^α^, p = 0.0031, t-test), and the SIR-nonSIR (5.0 Å for C^ω^ – C^ω^, p = 0.000019, t-test; 5.1 Å for C^α^ – C^α^, p = 0.000021, t-test; Table 3). As expected, the mean ∑∆SASA of the SIRPs was 168 Å^2^, which was significantly larger than that of the nonSIR-nonSIR (131 Å^2^, p = 0.000090, t-test) and the SIR-nonSIR (144 Å^2^, p = 0.0078, t-test). The mean ∑HE of the SIRPs was 6.2 [kcal/mol], which was significantly larger than that of the nonSIR-nonSIR (5.4 [kcal/mol], p = 0.00046, t-test), but not significantly different from that of the SIR-nonSIR (6.0 [kcal/mol], p = 0.25, t-test). The ∑∆Gi [kcal/mol] and DA[°] values were not significantly different among the SIRPs, nonSIR-nonSIR, and SIRP-nonSIR.

**Figure 2:**
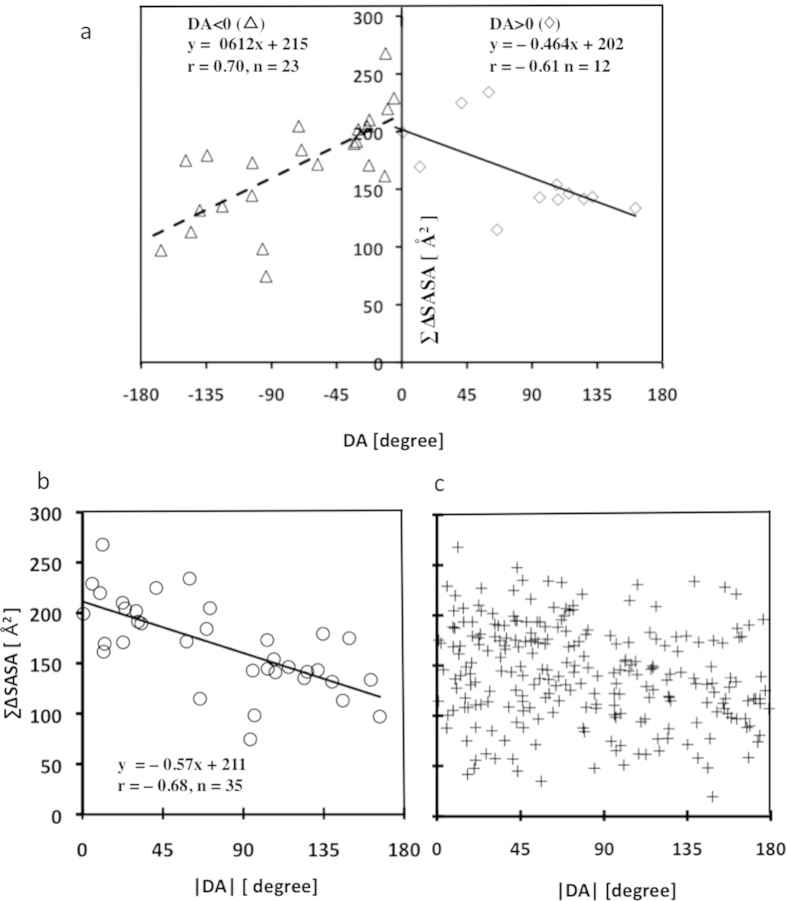
Correlations of the dihedral angles of the residues that were superimposed onto the inhibitors. (**a**) Correlation between DA and ∑∆SASA (DA>0 (n = 12, ◇) and DA<0 (n = 23, △)). (**b**) Correlation between |DA| and ∑∆SASA (n = 35, ○). (**c**) All 243 residue pairs (n = 243, +) were plotted on the graph (|DA| (*x*-axis) and ∑∆SASA (*y*-axis)).

Considering that visualizing the DA of four atoms (C^ω^ – C^α^ – C^α^ – C^ω^) and distances (C^α^ – C^α^, C^ω^ – C^ω^) is equivalent to the Sawhorse projections and the Newman projections in chemistry, we further investigated the correlation between shape-related descriptors (i.e., distances, DAs) and binding-related descriptors (i.e., hydrophobic interaction, ∆SASA, ∆G*i*) in the SIRPs and the non-SIRPs, and found strong correlations between the DA (*x*-axis) and ∑∆SASA (*y*-axis) in only the SIRPs (n = 35; Fig. 2a). Clear correlations between the DA and ∑∆SASA were observed for the positive DA values (DA > 0; r = −0.61, p < 0.035, n = 12) and negative DA values (DA < 0; r = 0.70, p < 0.00021, n = 23). Considering that the largest ∑∆SASA in both DA >0 and DA <0 increased as the DAs approached the zero degree (Fig. 2a), the absolute value of the DA (|DA|) was used instead of the DA (Fig. 2b). Once again, a clear correlation between |DA| and ∑∆SASA was observed (r = –0.68 with p < 0.00001, *y* = −0.57 *x* + 211, Fig. 2b). The correlation between |DA| and ∑∆SASA implied that not only ∑∆SASA (an interaction descriptor) but also |DA| (a shape-descriptor) can be used to distinguish SIRPs from non-SIRPs (Fig. 2b,c).

### Feasibility of the correlation using other inhibitors and another target protein

**Figure 3:**
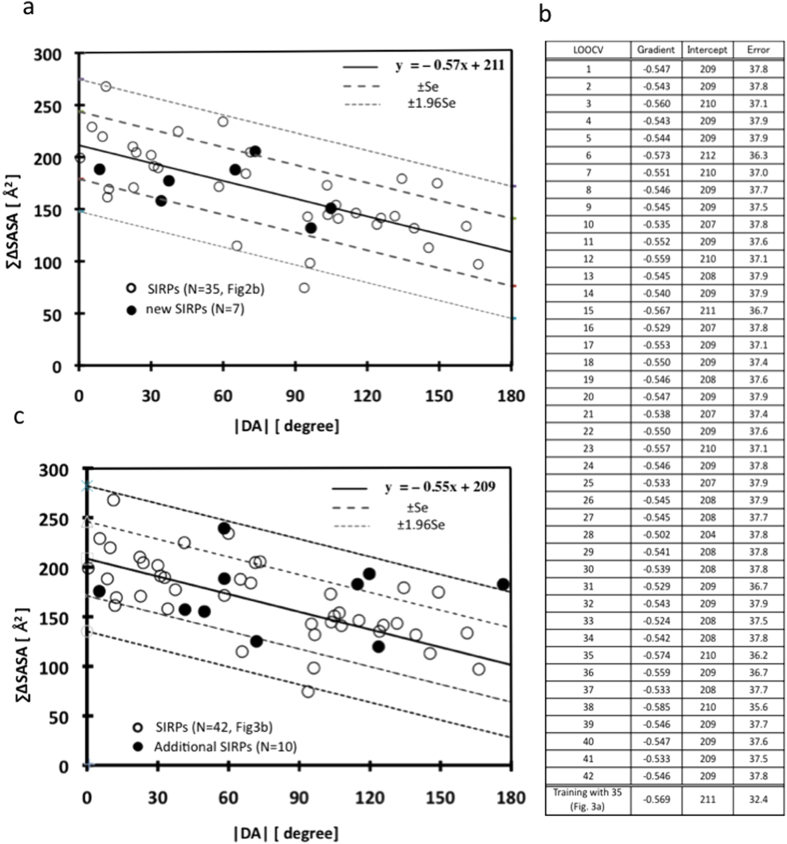
Application of the correlation between |DA| and ∑∆SASA (n = 35) (0). (**a**) The application of the correlation to 4 inhibitors that bind to new sites not bound by the 39 inhibitors(these 4 inhibitors bind to 3 of the 8 targets). Seven new SIRPs (●) are plotted in Fig. 2b. (**b**) LOOCV results using 42 samples (**c**) The application of the correlation to two novel targets (Keap1-Nrf2 and VHL-HIF1) and another MDM2 inhibitor,. 10 SIRPs (●) are plotted using the regression equation shown in Fig. 3b.

To demonstrate the feasibility of our hypothesis that the correlation between |DA| and ∑∆SASA could be useful for distinguishing SIRPs from non-SIRPs, we applied this correlation to an additional target-inhibitor dataset. First, we tested four additional inhibitors targeting three of the eight previously used target proteins (Supplementary Table 3). Notably, these inhibitors bind to different positions than the previous 39 inhibitors. From an inhibitor of BclxL (pdb: 4C52), we obtained three new SIRPs (I90–A91, I90–L94, and I90–I97)^35^. Two Mcl inhibitors (pdb: 4OQ5 and 4WGI) provided two additional SIRPs (I58–L62 and A59–L62)^36^,^37^. An integrase inhibitor (pdb: 3ZT1) provided two new SIRPs (K364–I365 and K364–D366)^38^. All seven new SIRPs were in the range of the regression equation ± SE (n = 35, ΣΔSASA = −0.57 *|DA|* + 211 ± SE, SE = 32.4; Fig. 3a). This finding suggests that the correlation can be used for new inhibitors, even when they bind to different positions on the same interfaces of their targets.

We then performed the Leave-One-Out Cross Validation (LOOCV) method using the 42 samples to properly and strongly validate the correlation between |DA| and ∑∆SASA because of the limited amount of data used. No statistically significant differences in the gradient or in the intercept of the regression equation were detected between the results of the 35 training samples and those of the LOOCV (Fig. 3b). Also, there was no difference between the estimated errors of the seven tested samples and those of the LOOCV, suggesting that the correlation between |DA| and ∑∆SASA was not incidental, but intrinsic.

We further tested the regression equation, which was based on the parameters obtained through the LOOCV process with 42 samples, using additional validation data of 10 new SIRPs, including novel target-partner protein combinations, such as Keap1/Nrf2 (pdb: 1×2R, Supplementary Table 4)^39^ and VHL/HIF1 (pdb:4AJY, Supplementary Table 4)^40^. Two Keap1 inhibitors (pdb: 4IQK, 3VNG) revealed four additional SIRPs (E79–T80, E79–E82, T80–E82, and E82–E83)^41^. Two new VHL inhibitors (pdb:4B9K, 3ZTC) resulted in three SIRPs (L562–A563, L562–I566, and A563–I566)^40^,^42^. A new Mdm inhibitor (pdb: 4LWV) resulted in three SIRPs (F19–L22, L22–W23, and L22–L26)^43^. These 10 SIRPs were plotted using the regression equation obtained from Fig. 3b (Fig. 3c). Of the 10 SIRPs, 9 were in the range of the regression equation ± 1.96 SE (n = 42, ∑∆SASA = −0.55 *|DA|* + 209 ± 1.96 SE, SE = 37.5), whereas the remaining SIRP was slightly out of range of the equation ± 1.96 SE. In addition, the distances of the 10 new SIRPs described above were shorter than the mean +1.96 SD for the 42 SIRPs (C^α^ – C^α^ 14.7 Å, C^ω^ – C^ω^ 16.0 Å), and the correlation between the |DA| and ∑∆SASA of all SIRPs (n = 52, r = −0.57, p = 0.00037, *y* = −0.47 *x* + 203, SE = 33.0) was nearly identical to the previous correlation (n = 42). This result suggests that the correlation could be applied to new inhibitors and unknown targets.

### The shortest SIRPs of each inhibitor can be used as a filter for extracting plausible SIRPs

The shortest SIRP distances (C^α^ – C^α^, C^ω^ – C^ω^) of the 48 inhibitors were selected to identify the distance necessary to inhibit the PPIs on the interfaces. Redundant SIRPs were removed, leaving 23 SIRPs (Supplementary Table 5). For these 23 SIRPs, the mean distances + 1.96 SD were 8.89 Å (C^α^ – C^α^) and 11.20 Å (C^ω^ – C^ω^) (C^α^: mean = 5.29 Å, SD = 1.84; C^ω^: mean = 7.22 Å, SD = 2.03). By contrast, the mean distances +1.96 SD for all 52 SIRPs were 14.3 Å (C^α^ – C^α^) and 16.1 Å (C^ω^ – C^ω^) (C^α^: mean = 7.16 Å, SD = 3.66; C^ω^: mean = 9.33 Å, SD = 3.56). These findings, including those of the LOOCV, validation with an additional dataset, and shorter distances, suggested that the correlation between the absolute value of the DA and the sum of the ∆SASAs of the residues could be applied to new inhibitors and unknown targets.

## Discussion

Considering that most of the SIRPs were non-polar residues and almost half (49%) were on α-helix motifs (Supplementary Fig. 2), we analyzed the effects of both the polarity and secondary structure to investigate whether the relation between |DA| and ∑∆SASA was intrinsic to SIRPs.

First, the residue pairs were classified into three groups: two non-polar residues (group 1); one non-polar residue and one polar residue (group 2); and two polar residues (group 3). The polar character of each group was in the order group1 < group2 < group3 (Supplementary Table 6). When the residue pairs were classified into three groups, group 3 had the smallest number of residue pairs and no SIRPs (nonSIR-nonSIR 14, SIR-SIR 8). Although there was no correlation between |DA| and ∑∆SASA between any of the nonSIR-nonSIR pairs, two SIRP groups showed correlations between |DA|(*x*-axis) and ∑∆SASA (*y*-axis) (group1: *r* = −0.67, p < 0.000014, n = 27, *y* = –0.55*x* + 212; group2: *r* = –0.54, p < 0.17, n = 8, *y* = −0.49*x* + 194). The slope and y-intercepts of groups 1 and 2 were similar to those of the pre-classification correlation, indicating that this correlation was not affected by differences in residue polarity. On the other hand, only one pair of SIR-nonSIR in group 3 showed a correlation between |DA| and ∑∆SASA (r = −0.75, p = 0.0319, n = 8, y = −0.584x+148). The difference between correlations of the SIRPs and the SIR-nonSIR pairs in group 3 was the y-intercept, indicating that polarity of the residue might affect the SIR-nonSIR pairs.

We then classified the residue pairs into nine groups, according to the combination of secondary structures between the residue pairs (Supplementary Fig. 2). There were eight combinations of secondary structures for non-SIRPs (n = 208) because none of the residue pairs were on different α-helices. When classified into the combinations, more than half of both the nonSIR-nonSIR and SIR-nonSIR pairs were on the same α-helix. One possible reason for this tendency is that an α-helix on the PPI interface is long enough to gain binding energy or ∑∆SASA from many residues that act as anchors on the interface.

Although there were no correlations between |DA| and ∑∆SASA for any of the secondary structure combinations of the non-SIRPs, SIRPs on the same α-helix (n = 17) and on the same loop or strand (n = 12) showed correlations between |DA| (*x*-axis) and ∑∆SASA (*y*-axis) (α-helix: *r* = −0.72, *p* < 0.0011, n = 17, *y* = −0.59*x* + 209; loop or strand: *r* = −0.43, *p* < 0.17, n = 12, *y* = −0.38*x* + 184). The slopes and y-intercepts of the two groups were also similar to those of the non-classified correlation. No other secondary structure combination was suitable for investigating the correlation because there were only two pairs of residues on the same β-turn and four pairs on the β-turn and loop (strand). These findings suggest that this correlation is not affected by different combinations of secondary structures to which the residues belong. Further study with large number of data should be performed to validate these findings.

Based on the definition of ΔG*i*^27^, it may be that ΔG*i* cannot explain the difference between SIRs and non-SIRs (Table 2). We think, however, that there may be no difference in ΔG*i* between SIRs and non-SIRs because we did not use ΔG*i* to select the SIRs. Instead, by focusing on the SIRs, we noticed that the ΔSASA of SIRs was different from that of the non-SIRs, leading to further studies of the ΣΔSASA of SIRPs.

**Figure 4:**
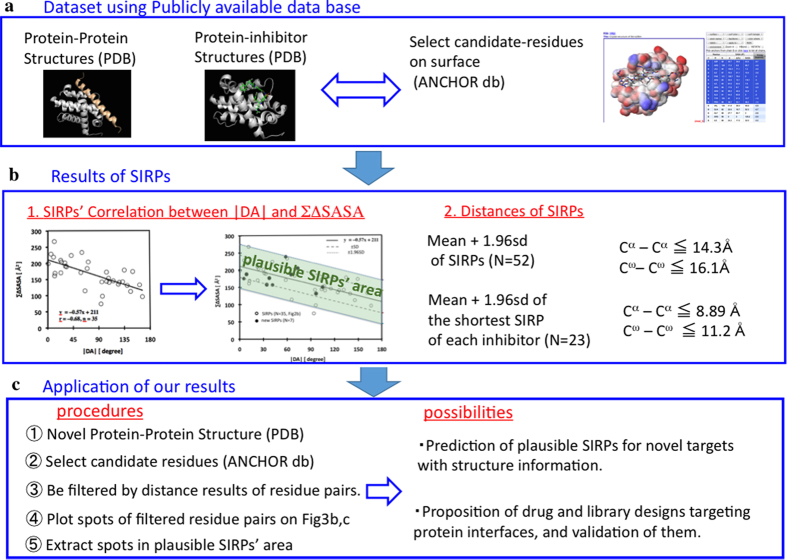
Scheme summarizing the application of our results.

Recently, Moreira and colleagues reported a method for predicting hot spots in protein-protein and protein-nucleic acid interfaces based on the SASA)^44^, which is consistent with a previous report demonstrating that ∆SASA is important to anchor residues^27^. Our results, extracted utilizing three publicly available databases, demonstrated that features of SIRPs correlated between |DA| and ∑∆SASA and the distances between residue pairs (Fig. 4a,b). These findings could be applied to novel inhibitors and a novel target (Fig. 4c). One example of the application is to filter out non-SIRPs and select plausible SIRPs for novel targets (noted in Supplementary information, Supplementary Table 7).

Although we determined the contribution of the residue using the ANCHOR database, there are other published methods for determining the contribution of a residue at a PPI, such as Rosetta scanning^45^ and mCSM-PPI^46^. Therefore, we used the mCSM-PPI method with a single mutation. When the 64 residues shown in Table 2 were mutated to alanine, we observed a slight but nonsignificant difference between SIRs (n = 26, mean ∆∆G −1.687 kcal/mol SD 0.845) and non-SIRs (n = 34, mean ∆∆G −1.286 kcal/mol SD 0.858; p = 0.071). Further studies are needed to determine the contribution of a residue at a PPI.

To validate the feasibility of our regression model against the data that were either out of our criteria or out of our selection procedure mentioned before, we tested the regression equation using additional data of 17 SIRPs, including another target-partner protein combination, such as IL2/IR2R (pdb: 1Z92)^47^, and family proteins, such as, cIAP1-BIR3/Smac (pdb: 3D9U) and Bcl2/BAX (pdb: 2XA0) (Supplementary Table 8). Two IL2 inhibitors (pdb: 1M49, 1PY2), which were reported in 2003, resulted in two SIRPs (L2-R36, D4-R36)^48^,^49^. Also as mentioned in the “RESULTS” section, the following four inhibitors were NOT selected as the original 39 protein-inhibitor complexes in order to avoid redundancy. Two selective cIAP-1 inhibitors (pdb: 4LGU, 4LGE) resulted in 6 SIRPs (A1-V2, A1-P3, A1-I4, V2-P3, V2-I4, and P3-I4)^50^,^51^. Two selective Bcl2 inhibitors (pdb: 4LVT, 2W3L) resulted in 9 SIRPs (L59-L63, L59-C62, C62-L63, L63-L70, L63-D71, L63-M74, L70-D71, L70-M74 and D71-M74)^52^,^53^. By applying our regression equation, which was based on the parameters obtained through the LOOCV process, 17 SIRPs were in the range of the regression equation ± 1.96 SE (n = 42, ∑∆SASA = −0.55 *|DA|* + 209 ± 1.96 SE, SE = 37.5) (Supplementary Figure 3). In addition, the distances of the 15 additional SIRPs described above were shorter than the mean +1.96 SD for the 42 SIRPs (C^α^ −C^α^ 14.7 Å, C^ω^ −C^ω^ 16.0 Å), except for 2 SIRPs of IL2. The correlation between the |DA| and ∑∆SASA of all SIRPs (n = 69, r = −0.533, p < 0.00001, *y* = −0.419 *x* + 199, SE = 32.6) was nearly identical to the previous correlation (n = 42).

In summary, we focused on two-residue relationships and found a linear negative correlation between |DA| and ∑∆SASA for SIRPs based on a comparison of the protein-protein complexes and the protein-inhibitor complexes. This correlation was successfully applied to five additional inhibitors of different targets. Our results shed light on the two-residue correlation between the absolute value of the DA and the sum of the ∆SASAs. Further studies should be performed to evaluate multi-residue correlations by focusing on three- (or more) residue relationships.

## Methods

### Data collection

The set of complex structures evaluated in this study is listed in Table 1. The structures of the complexes were obtained from the PDB and TIMBAL. All structural figures were generated using PyMOL (http://www.pymol.org). The predicted values of ∆SASA and ∆G*i* are publicly available from the ANCHOR database (http://structure.pitt.edu/anchor)^27^,^29^,^30^. The ∆SASA for each side-chain upon binding and an estimate of its contribution to the ∆G*i* are listed in the database. Rajamani *et al.* calculated the conformation-dependent portion of the empirical ∆G*i* using the expression ∆G*i* = ∆ E_elec_(*i)* +∆*Gdes*(*i)*, where ∆E_elec_(*i)* denotes the electrostatic interaction energy between atoms in the ligand residue *i* and the receptor, and ∆*Gdes*(*i)* is an estimation of the desolvation free energy of residue *i*^27^, To estimate the hydrophobic interactions of each selected residue, we used the estimated values for the HE of the amino acid residues reported by Karpus^54^. These data for the 64 selected residues are summarized in Supplementary Table 1. Polar and non-polar side chains of amino acids were classified by Perutz’s method^55^.

### Determination of the superimposed residues

Every residue that was superimposed onto an inhibitor (SIR) was selected using the same method used for the Mcl/p53 complex (PDB:1YCR) and a corresponding protein-inhibitor complex (PDB:1RV1), which is shown as an example in Supplementary Fig. 1. First, we used the “align” command in PyMOL to perform structural alignments between the structures of the target protein in the native protein–protein complexes and the structures of the corresponding protein-inhibitor complexes (Supplementary Fig. 1a). The native sequence of the target protein and the corresponding sequence of the inhibitor–bound target protein were considered to have few differences. The average root-mean-square deviation was 0.866 Å (Supplementary Table 9). The bonds of the selective residues of the partner protein were then drawn as sticks, whereas the inhibitors were drawn as spheres with radii equal to the van der Waals radii (Supplementary Fig. 1b,c). Finally, when a residue containing at least two heavy atoms (other than the atoms of the amide bond) whose centers were superimposed onto the sphere of the inhibitor was observed, the residue was considered to be superimposed onto the inhibitor and was thus defined as a SIR. Residue pairs were determined in the same way. After the redundant residue pairs were removed, 35 residue pairs that were superimposed onto 39 inhibitors were found (Supplementary Table 10).

### Structure of the residue pairs

In this report, every combination of two residues on the same drug target was defined as a residue pair. The total number of residue pairs was the sum of the combination of *n* selected residues taken two at a time in each target (∑{_n1_C_2_ (target1) + _n2_C_2_ (targt2) + _n3_C_2_ (target3) + ^…^ + _n8_C_2_ (target8)} = 243 [residue pairs]). A total of 243 residue pairs were found in the partner proteins of those proteins targeted by the 8 drugs. PyMOL was used to measure the distances and DAs between the residue pairs. The distance between the C^α^ (alpha carbon of an amino acid) of one residue and C^α^ of the second residue (C^α^ – C^α^) was measured for each residue pair. The distance between the C^ω^ (the farthest carbon atom from C^α^ or C^β^ carbon of an amino acid) of one residue and the C^ω^ of the second residue (C^ω^ – C^ω^) was also measured. The C^ω^ – C^α^ – C^α^ – C^ω^ DAs of each residue pair were also measured. Basically, C^ω^ was defined as either the farthest heavy atom from the C^β^ of the side chain of an aromatic amino acid or the end heavy atoms of the side chain of a non-aromatic amino acid. For the branched end amino acids (Val, Leu, Glu, Gln, Asp, Asn, and Arg), the carbon atoms that branched before the end atoms were assumed to be the farthest atoms (C^ω^). With Pro, C4 was assumed to be the farthest atom from C^α^. The sum of HE (∑HE), ∆G*i* (Σ∆G*i*), and ∆SASA (∑∆SASA) for each residue pair was calculated. All residue pairs (n = 243) were investigated (Supplementary Table 2).

### Statistical analysis

Two-tailed Student’s t-test assuming equal variances was used in this study to compare the mean ΔSASA and ΔΔG of the SIRs vs. non-SIRs as well as to compare the mean distances between the atoms (C^ω^ – C^ω^, C^α^ – C^α^), ΣΔSASA and ΣHE of the SIRPs vs. nonSIR-nonSIR and SIR-nonSIR. The Pearson correlation coefficient was calculated to measure the linear relationship between DA and ΣΔSASA as well as between |DA| and ΣΔSASA. Two-tailed p-value for the correlation coefficient was calculated using Student’s t-distribution.

## Additional Information

**How to cite this article**: Nakadai, M. *et al.* An Intriguing Correlation Based on the Superimposition of Residue Pairs with Inhibitors that Target Protein-Protein Interfaces. *Sci. Rep.* **6**, 18543; doi: 10.1038/srep18543 (2016).

## Supplementary Material

Supplementary Information

## Figures and Tables

**Table 1:** Eight target-partner proteins, 39 inhibitors, and 64 selected residues.

Target protein/partner protein	PDB IDs of protein-protein complexes	PDB IDs of protein-inhibitor complexes	Selected residues (n = 64)	
			superimposed residues (n = 26)	non-superimposed residues (n = 38)
BCL-xL/Bim	1PQ1	1YSI, 3ZLN, 2YXJ, 3ZLR, 3SP7	A91, L94, I97, D99, F101, Y105	R85, P86, R89, I90, R95, R108
Integrase/LEDGFp75	2B4J	3LPT, 3LPU, 3ZSO, 4LH5, 4ELM, 4E1N	I365, D366, L368	K360, K364, F406, V408
Mcl/Bim BH3	2NL9	4HW2, 3WIX	L62	E55, I58, A59, R63, I65, D67, F69, Y73
Menin/MLL	4GQ6	4GQ3, 4OG4, 4OG3	F9, P10, P13	A5, R6, W7, R8, A11, R12
Mdm/p53	1YCR	4JV9, 4JVE, 4J7D, 4J7E, 4IPF, 1RV1, 1T4E, 3JZK, 3LBK, 3LBL, 4ERE, 2LZG	F19, W23, L26	E17, L22, P27, E28,
XIAP-BIR3/Caspase 9	1NW9	1TFQ, 1TFT	A316, T317, P318, F319	L244, L385, C403, F406, K410
XIAP-BIR3/Smac	1G73	3EYL, 2OPY, 4HY0, 4KJV, 3GTA, 3F7I	A1, V2, P3, I4	—a
ZipA/Fits	1F47	1S1S, 1Y2F, 1Y2G	F11, L12	D4, Y5, L6, D7, I8

^a^There were no non-superimposed residues in XIAP-BIR3/Smac.

**Table 2:** Difference between the superimposed residues^a^ (n = 26) and non-superimposed residues^b^ (n = 38).

		all residues (n = 64)	superimposed residues^a^ (n = 26)	non-superimposed residues^b^ (n = 38)
Secondary structures^c^		number	number	number
	α-Helix	34	12	22
	β-turn	9	4	5
	loop^d^	21	10	11
		**Mean (median) SD**	**Mean (median) SD**	**Mean (median) SD**
Descriptors	ΔG*i* [kcal/mol]	−3.5 (−3.0) 2.5	−3.6 (−3.1) 2.2	−3.5 (−2.6) 2.8
	HE [kcal/mol]	2.9 (3.1) 0.9	3.2 (3.5) 0.9	2.7 (2.3) 0.9
	ΔSASA [Å^2^ ]	71.8 (73.4) 34.5	85.4 (86.2) 33.1	61.9 (62.5) 32.5

^a^Residues that were superimposed onto the inhibitors.

^b^Residues that were not superimposed onto the inhibitors.

^c^Number of secondary structures to which the residues belonged.

^d^Loop or strand.

**Table 3:** Comparison among the superimposed residue pairs^a^, non-superimposed residue pairs^b^, and superimposed residue-non-superimposed residue pairs^*c*^.

	All residue pairs (n = 243)	Superimposed residue pairs (SIRPs)^a^ (n = 35)	nonSIR-nonSIR^b^ pairs (n = 90)	SIR-nonSIR^c^ pairs (n = 116)
	Mean (median) SD	Mean (median) SD	Mean (median) SD	Mean (median) SD
distance (C^ω^- C^ω^) [Å]	13.3 (11.9) 6.4	9.3 (8.7) 3.9	13.7 (12.7) 6.7	14.3 (13.1) 6.3
distance (C^α^ – C^α^) [Å]	11.4 (10.1) 6.5	7.6 (6.9) 4.0	11.0 (9.9) 6.8	12.7(12.1) 6.8
∑ΔSASA [Å ^2^]	141.8 (141.0) 48.2	167.8 (170.8) 42.8	130.5 (125.5) 47.1	143.3 (145.9) 47.1
∑ΔG*i* [kcal/mol]	−6.9 (−6.2) 3.5	−7.2 (−6.5) 3.0	−7.3 (−6.2) 4.1	−6.4 (−5.9) 2.9
∑HE [kcal/mol]	5.8 (5.7) 1.2	6.2 (6.3) 1.1	5.4 (5.3) 1.2	6.0 (5.8) 1.2
DA [degree]	−13.7 (−14.1) 93.7	−18.1 (−22.7) 90.2	−19.0 (−15.7) 97.9	−9.5(−6.3) 92.0
|DA| [degree]	79.6 (70.8) 51.3	76.8 (71.3) 51.0	83.3 (84.0) 54.9	78.0 (75.8) 46.2

^a^Pairs of residues that were superimposed onto the inhibitors.

^b^Pairs of residues that were not superimposed onto the inhibitors.

^c^Pairs that one residue was superimposed onto an inhibitor and another was not superimposed onto inhibitors.
