# Supplementary material for: An Intriguing Correlation Based on the Superimposition of Residue Pairs with Inhibitors that Target Protein-Protein Interfaces
Source: Sci Rep. 2016 Jan 4;6:18543. doi: 10.1038/srep18543 (PMC4698585; doi:10.1038/srep18543)
Supplement: Supplementary Information [file srep18543-s1.doc]

An Intriguing Correlation Based on the Superimposition of Residue Pairs with Inhibitors that Target Protein–Protein Interfaces.

AUTHOR NAMES

Masakazu Nakadai*, Shuta Tomida, Kazuhisa Sekimizu*

Supplementary Imformation

Supporting text

Supplementary Table 1: 64 single residues.

Supplementary Table 2: all data for 243 residue pairs (35 SIRPs).

Supplementary Table 3: 7 additional SIRPs from 4 inhibitors for LOOCV.

Supplementary Table 4: Residue pairs of Keap1/Nrf2 and VHL/HIF1 (7 additional SIRPs of the 10)

Supplementary Table 5: The shortest SIRP distances of the 48 inhibitors.

Supplementary Table 6: a comparison of the residue pair data that were classified into three groups on the basis of polarity.

Supplementary Table 7: one example of the application of the correlation to Keap1-Nrf.

Supplementary Table 8: Residue pairs of IL2/IL2R, cIAP1-BIR3/smac and Bcl2/Bax (additional 17 SIRPs)

Supplementary Table 9: All structural data (PDB) for 243 residue pairs

Supplementary Table 10: Residue pairs (n=35) that were superimposed onto 39 inhibitors.

Supplementary Figure 1: An example of how the superimposition of residue pairs with an inhibitor can be defined.

Supplementary Figure 2: the classification of the residue pairs (n=243).

Supplementary Figure 3: 17 additional SIRPs spotted on Figure 3c

This material is available free of charge via the Internet at <http://www.nature.com/srep>.

**Supporting Text**

**Results**

**How to count the number of residue pairs**

Theoretically, the sum of the number of SIRPs should be 37 (∑{_6_C_2_ (Bcl-xL) + _3_C_2_ (Integrase) + 0 (Mcl: 1SIR, 0 SIRP) + _3_C_2_ (Menin) + _3_C_2_ (Mdm) + _4_C_2_ (XIAP(caspase9)) + _4_C_2_ (XIAP(smac) + _2_C_2_ (ZipA)) = 37 [residue pair]}. However 35 SIRPs were found from 39 inhibitors (Supplementary Table 10). The number of SIR-SIR residue pairs that no inhibitor was superimposed with was two.

**Discussion**

**Application for the correlation to extract plausible SIRPs**

We considered one explanation of the application for keap1-Nrf2 (Supplementary Table 7). First, we selected 7 candidate­ residues from ANCHOR db. A pair of two residues could be selected from a pair of 7 residues in _7_C_2_ = 21 ways. Second, when 21 residue pairs were filtered through the shortest SIRPs’ filters (Cα – Cα 8.89 Å , Cω – Cω  11.2 Å ), we could extract 13 residue pairs as candidates of plausible SIRPs. Third, when we spotted the 13 residue pairs on the regression equation of the correlation between |DA| and ∑∆SASA, the number of the spot in the range of the regression equation ± 1.96 S.E. was 10, which we called ‘plausible SIRPs’. All SIRPs of the Keap1 inhibitors (pdb: 4IQK, 3VNG) were plotted in the range. Forth, the number of the spot in the range of the equation ± S.E. was 5. The two of four SIRPs of the Keap1 inhibitor were plotted in the range.

**Supplementary Table 1: 64 single residues.**

**
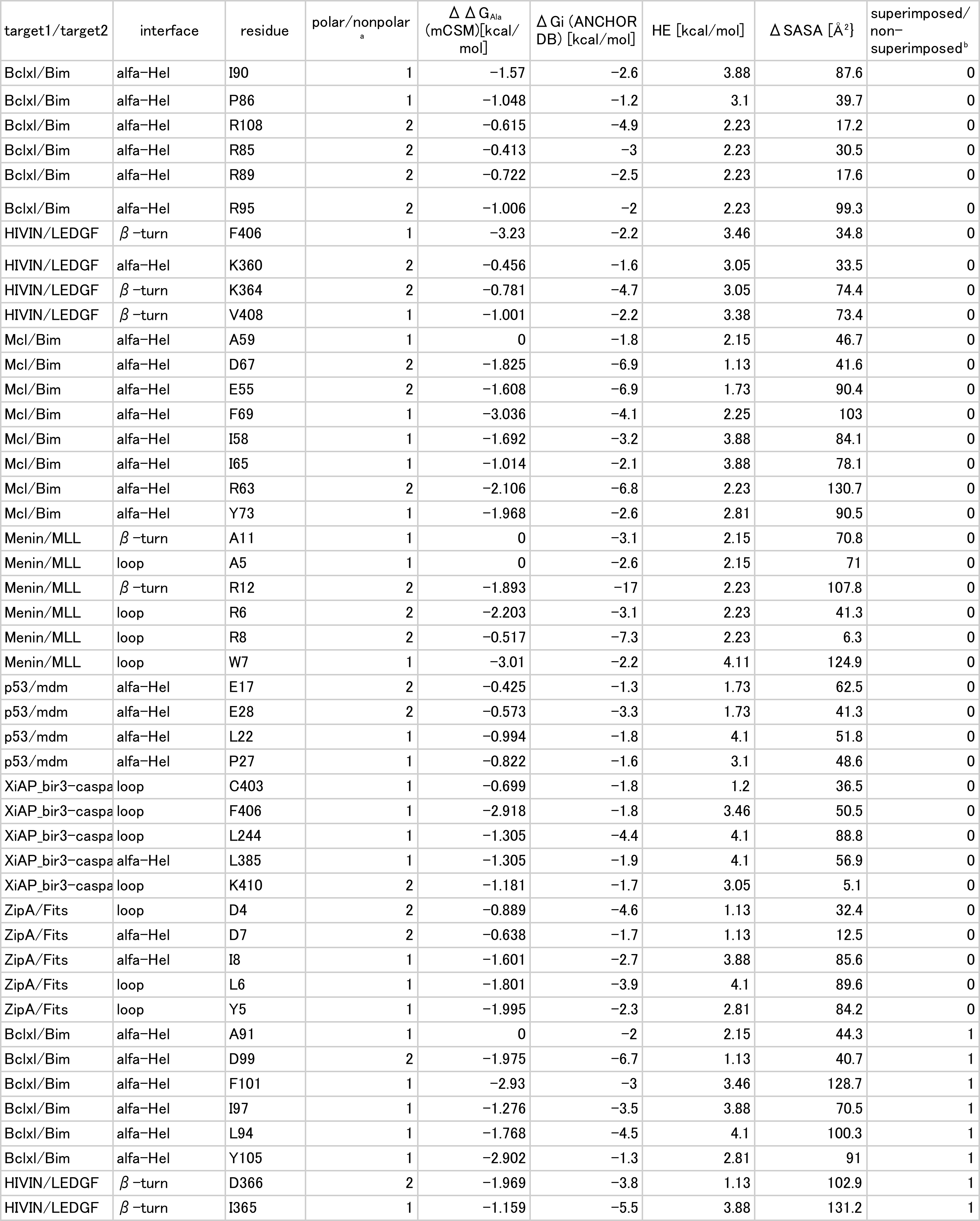
**


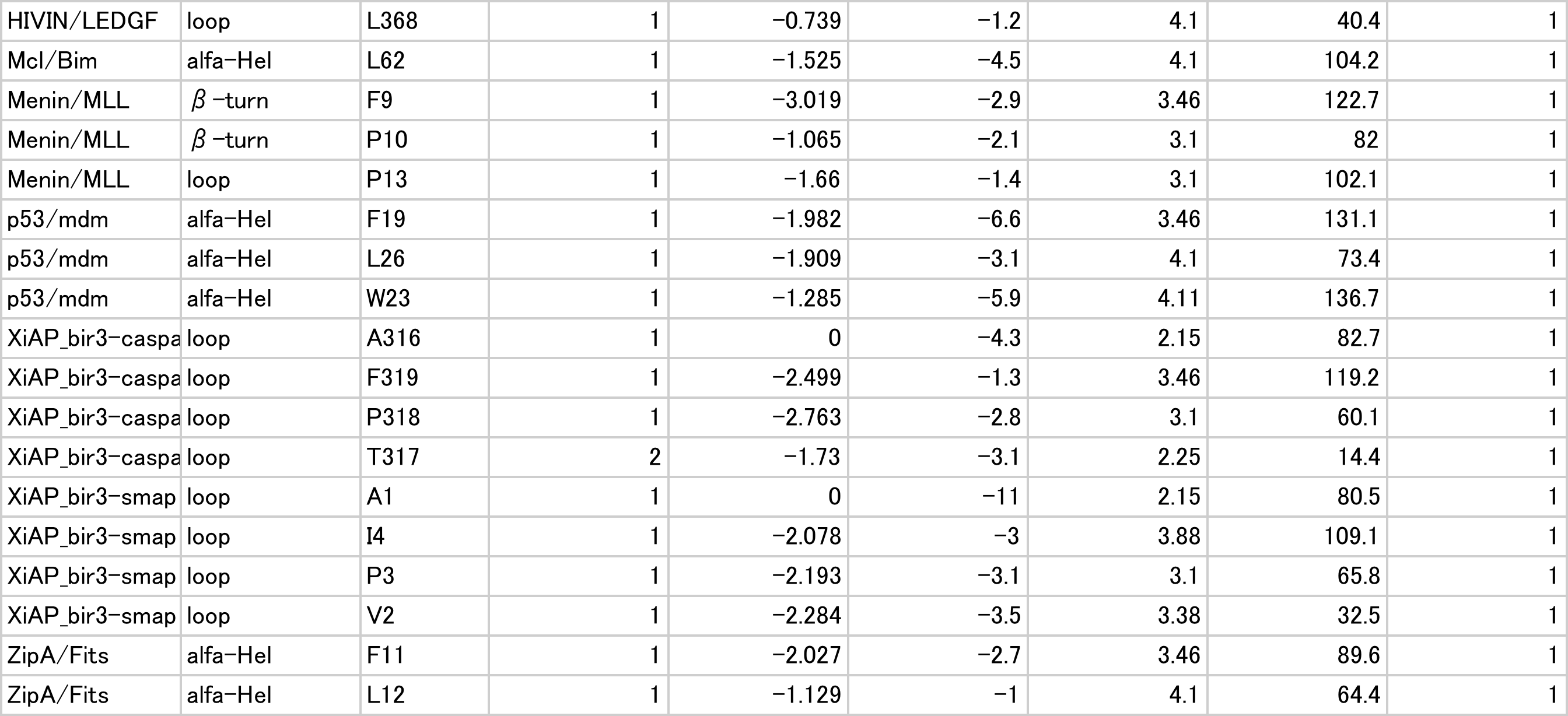


^a^ 1: non-polar residues, 2: polar residues (*J.Mol.Biol*. **13**, 669-678 (1965**)**, Perutz et.al)

^b^ 0: residues that were non-superimposed with inhibitors, 1: residues that were superimposed with inhibitor

**Supplementary Table 2: All data for 243 residue pairs.**

**
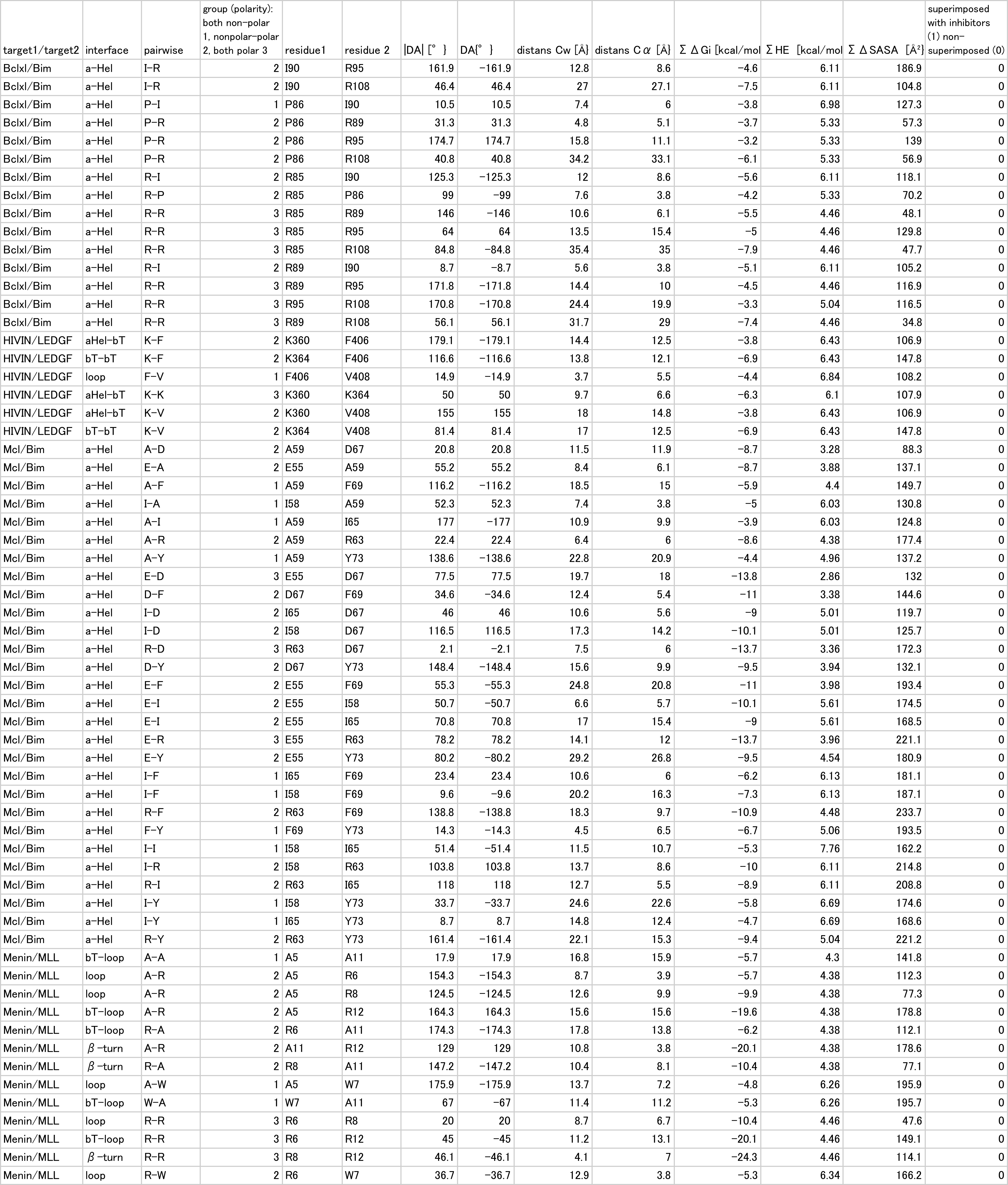
**


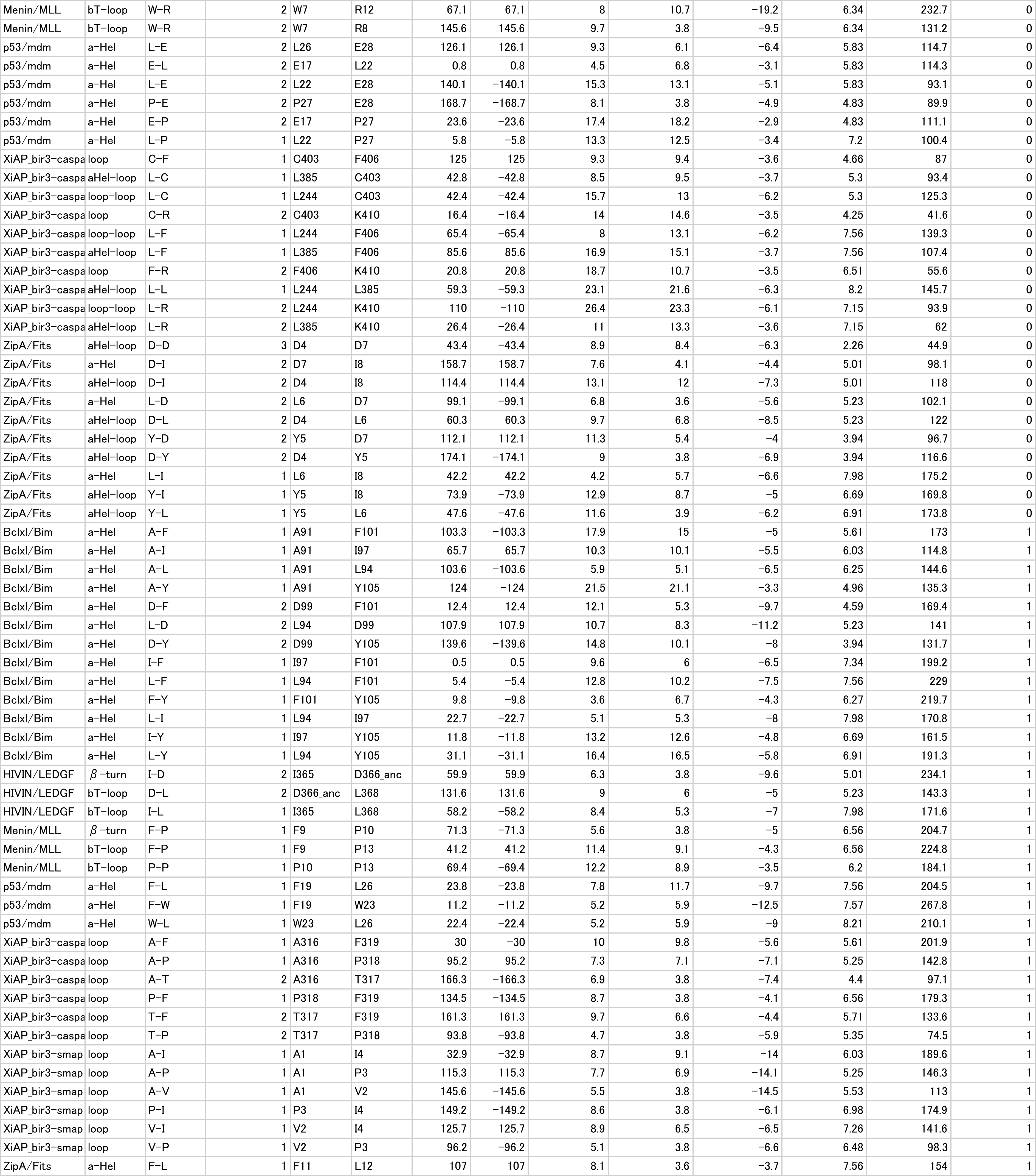


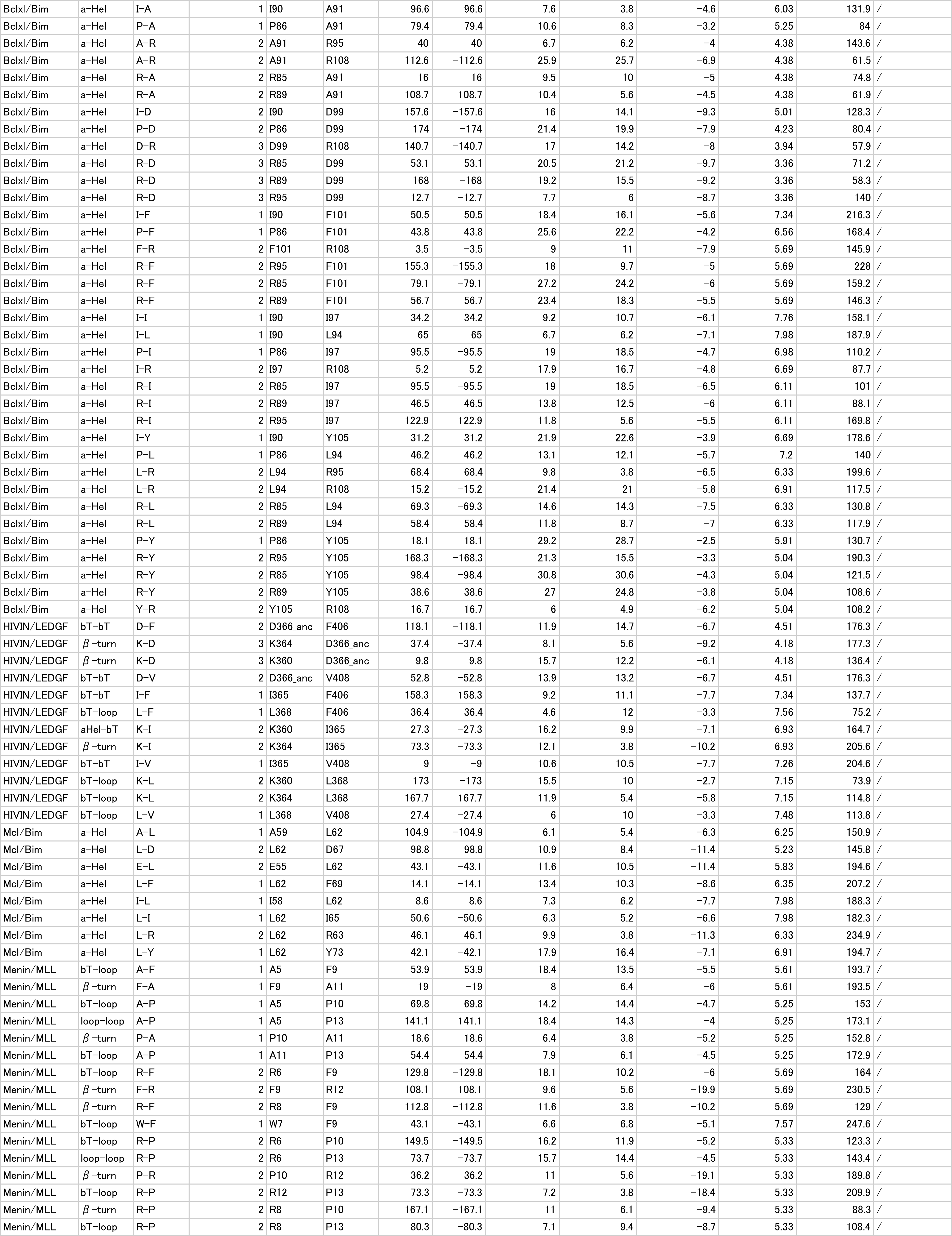


a
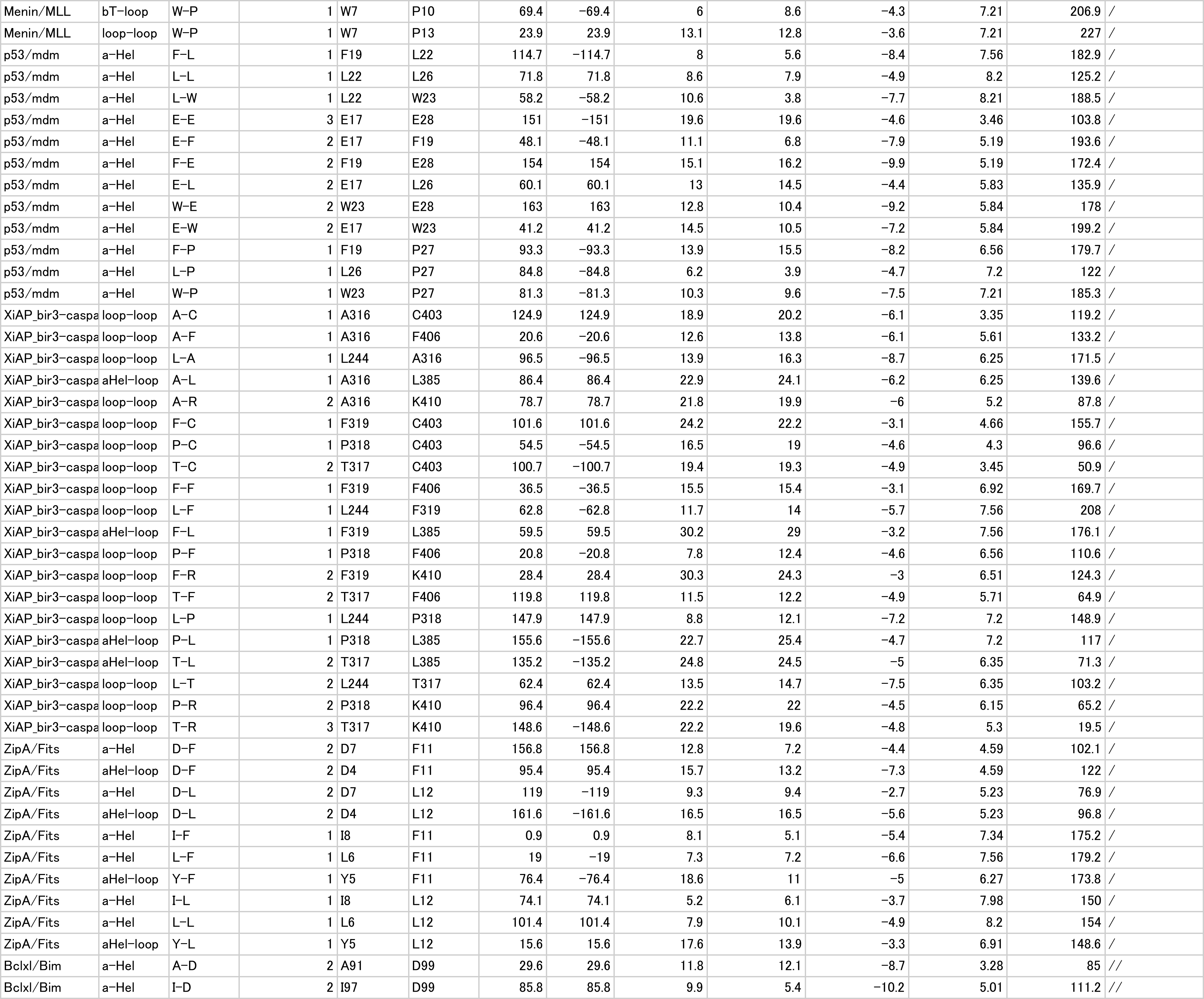
 The right column: SIRPs n=35 (1), nonSIR-nonSIR pairs n=90 (0), SIR-nonSIR pairs n=116 (/), SIR-SIR pairs (no inhibitor that was superimposed with both residues.) n=2 (//)

**Supplementary Table 3. 7 additional SIRPs from 4 inhibitors for LOOCV. (Figure 3a, b)**


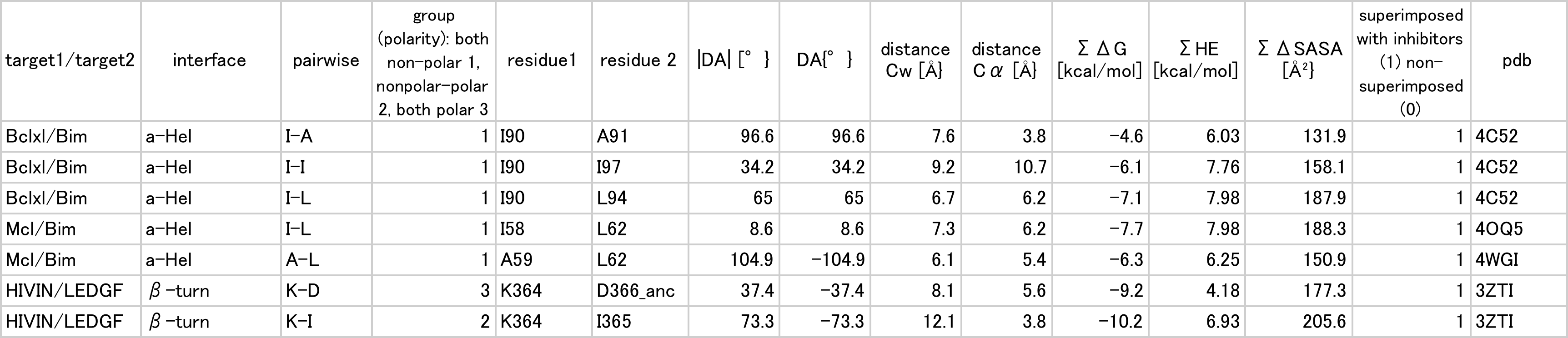


**Supplementary Table 4: Residue pairs of Keap1/Nrf2 and VHL/HIF1.**

**
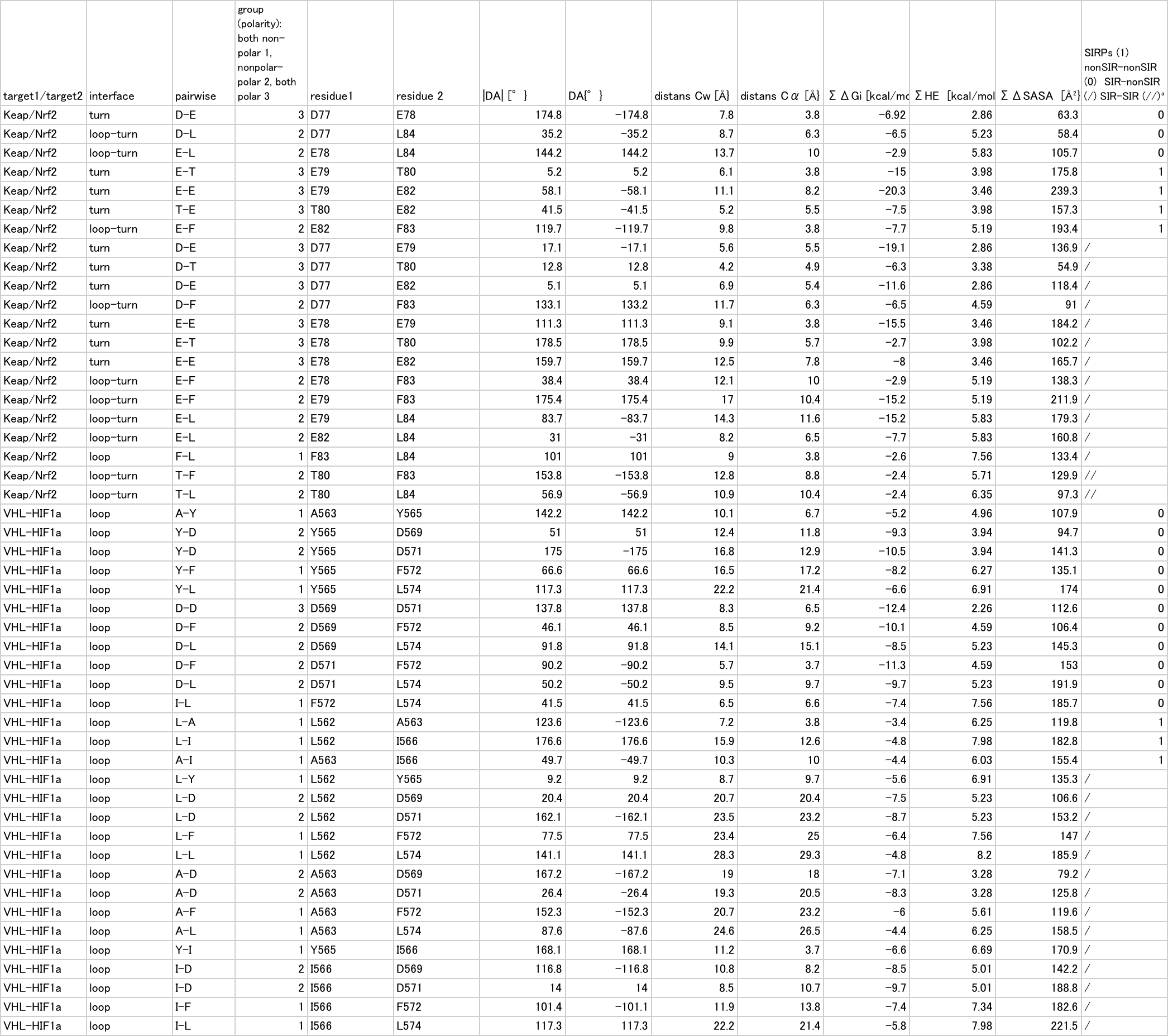
**

^a^ The right column: SIRPs n=35 (1), nonSIR-nonSIR pairs n=90 (0), SIR-nonSIR pairs n=116 (/), SIR-SIR pairs (no inhibitor that was superimposed with both residues.) n=2 (//)

^b^ Two VHL inhibitors (pdb: 4B9K, 3ZTC) were superimposed on Hyp264. However, residue pairs that contained Hyp264 were not contained in this table, because unusual amino acids are not able to calculate the values (∆SASA and –∆G*i*) using ANCHOR database.

**Supplementary Table 5: The shortest SIRP distances of the 48 inhibitors.**


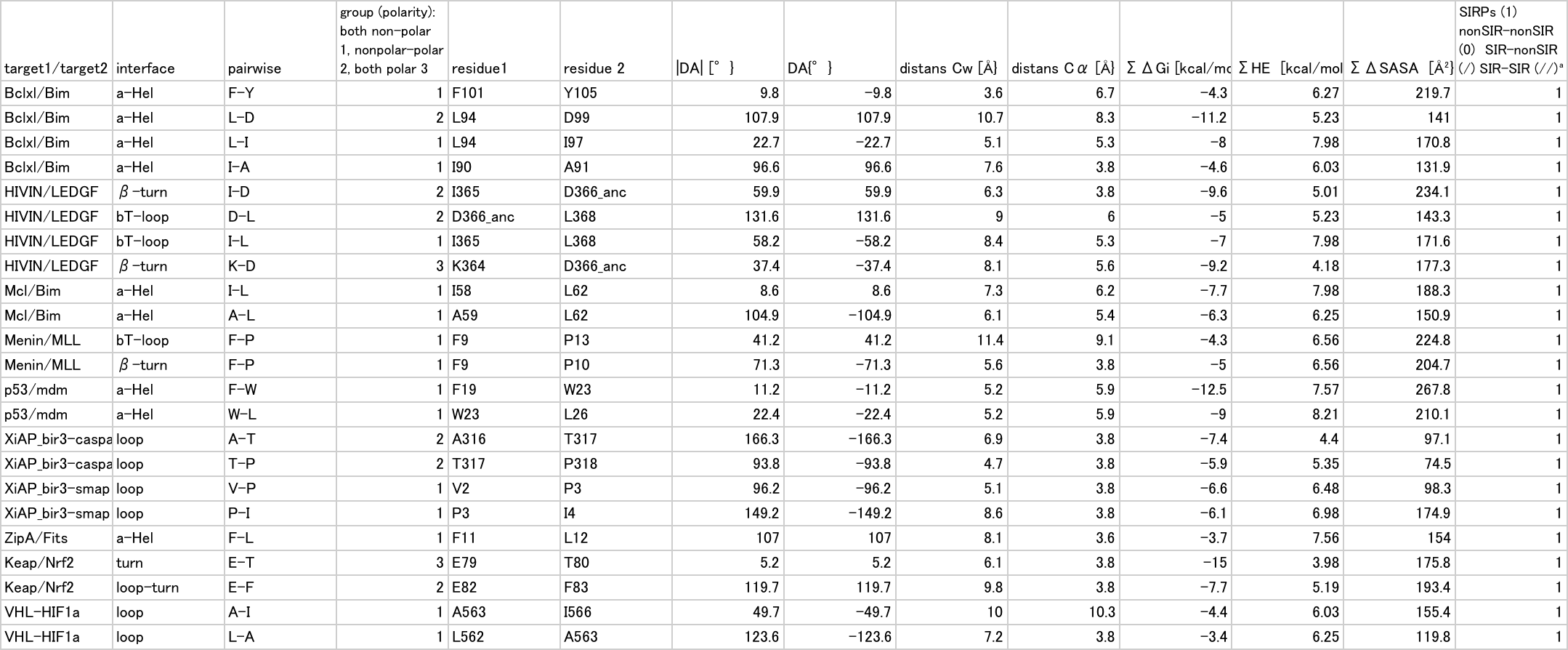


**Supplementary Table 6:** **A comparison of the residue pair data that were classified into three groups on the basis of polarity**


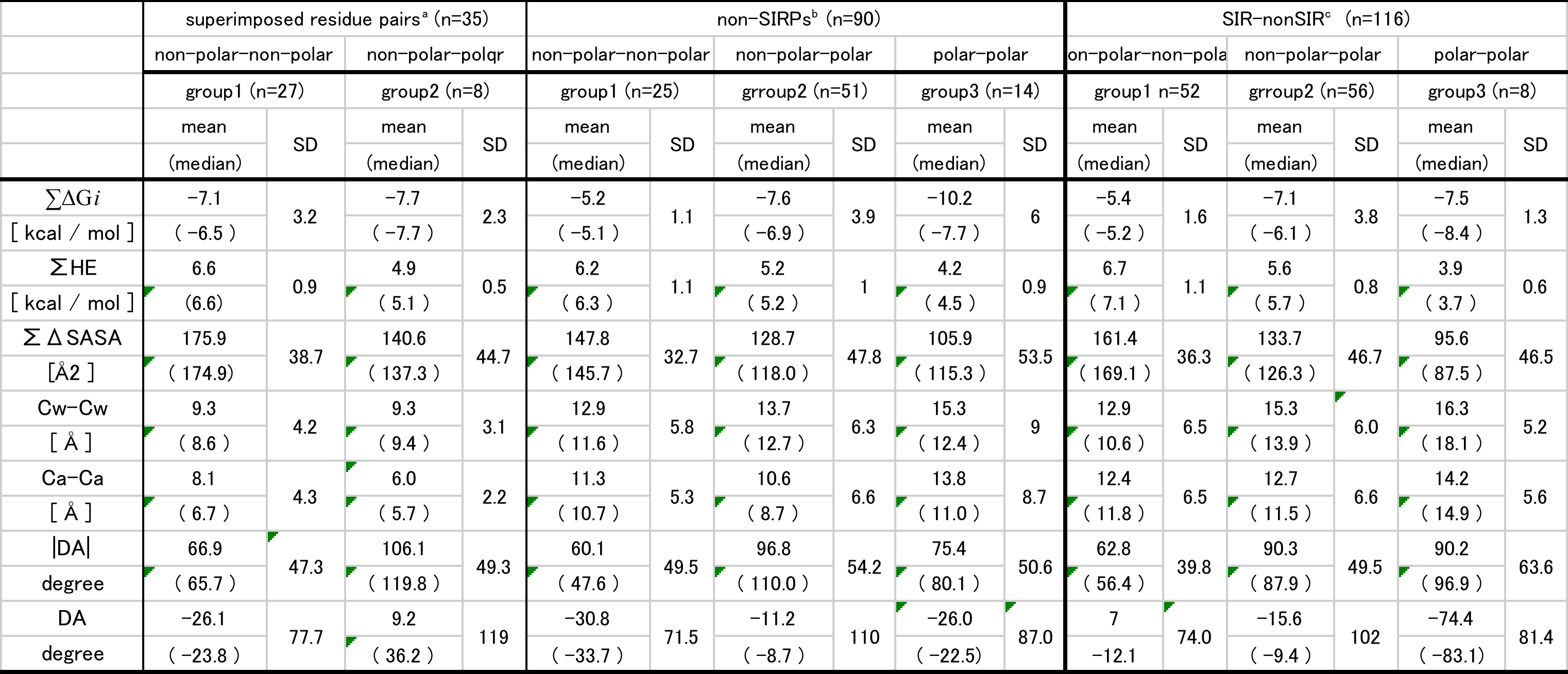


^a^ Pairs of residues, which are superimposed onto small molecules.

^b^ Pairs of residues, which are not superimposed onto small molecules.

*^C^* Pairs that one residue was superimposed onto an inhibitor and another was not superimposed onto inhibitors.

**Supplementary Table 7: One example of the application of the correlation to Keap1-Nrf2**

| Filter | - | - |  | the regression of the colleration^b^ | |
| --- | --- | --- | --- | --- | --- |
|  | all | distances^a^ |  | ±1.96se | ±se |
| the number of residue pairs | 21 | 13 |  | 10 | 5 |
|  |  |  |  |  |  |
| List of plausible SIRPs after filtration | | |  |  |  |
| plausible SIRPs | ±1.96se | D77-F78, D77-E79, E78-E79, E78-T80^*^, E79-E82^*^, T80-E82^*^, E82-L84, E82-F83^*^, F83-D77 F83-L84, | | | |
|  |  |  |  |  |  |
|  | ±se | E78-T80, E79-T80^*^, T80-E82^*^, E82-L84, F83-L84 | | | |

* identified as SIRPs (pdb: 4IPQ, 3VNG).

^a^ Cα – Cα ≤ 8.9 Å, Cω – Cω ≤ 11.2 Å

^b^ *ΣΔSASA*= – 0.55 *|DA|* + 209, se=37.5

**Supplementary Table 8: Residue pairs of IL/IL2R, cIAP1-BIR3/smac and Bcl2/Bax.**

**
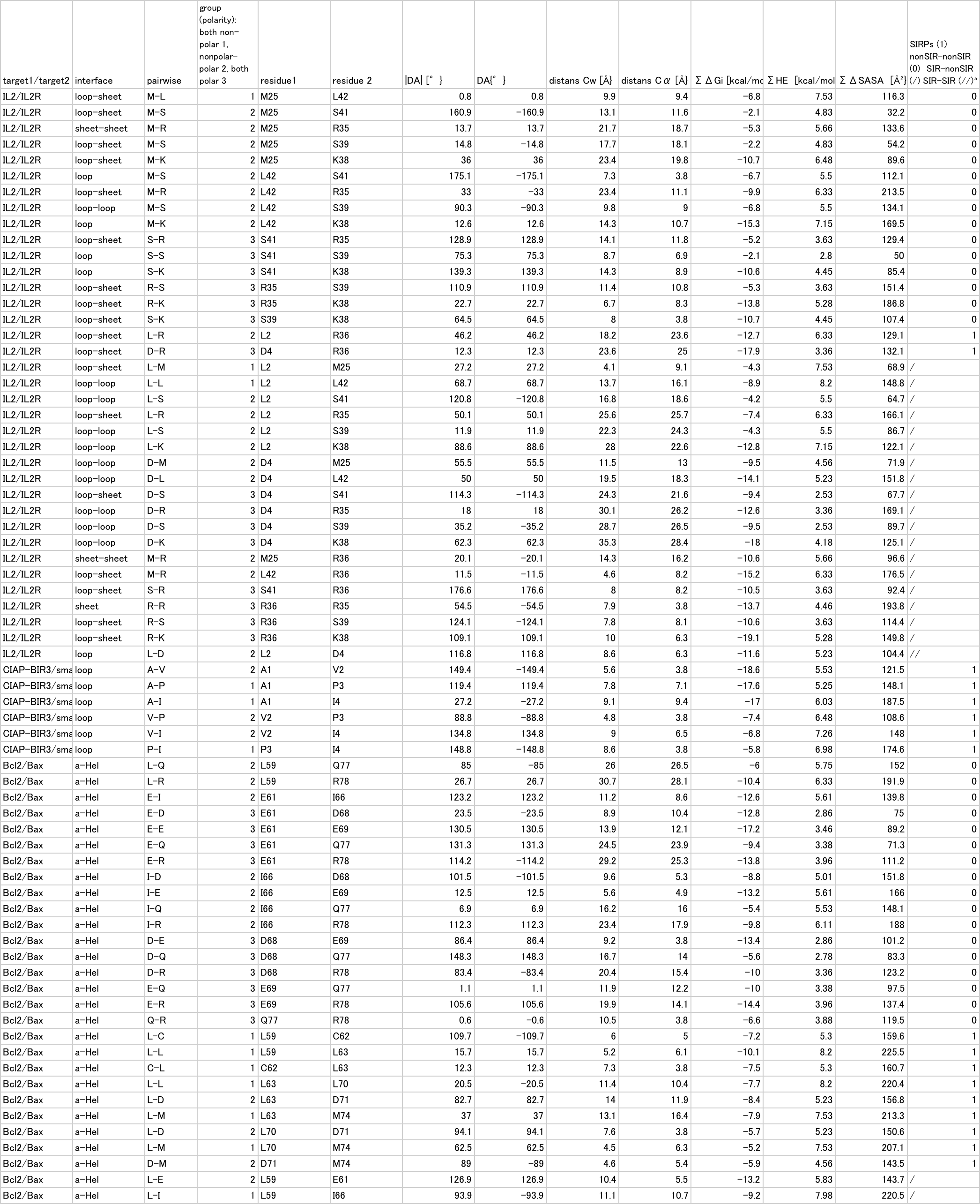
**

**
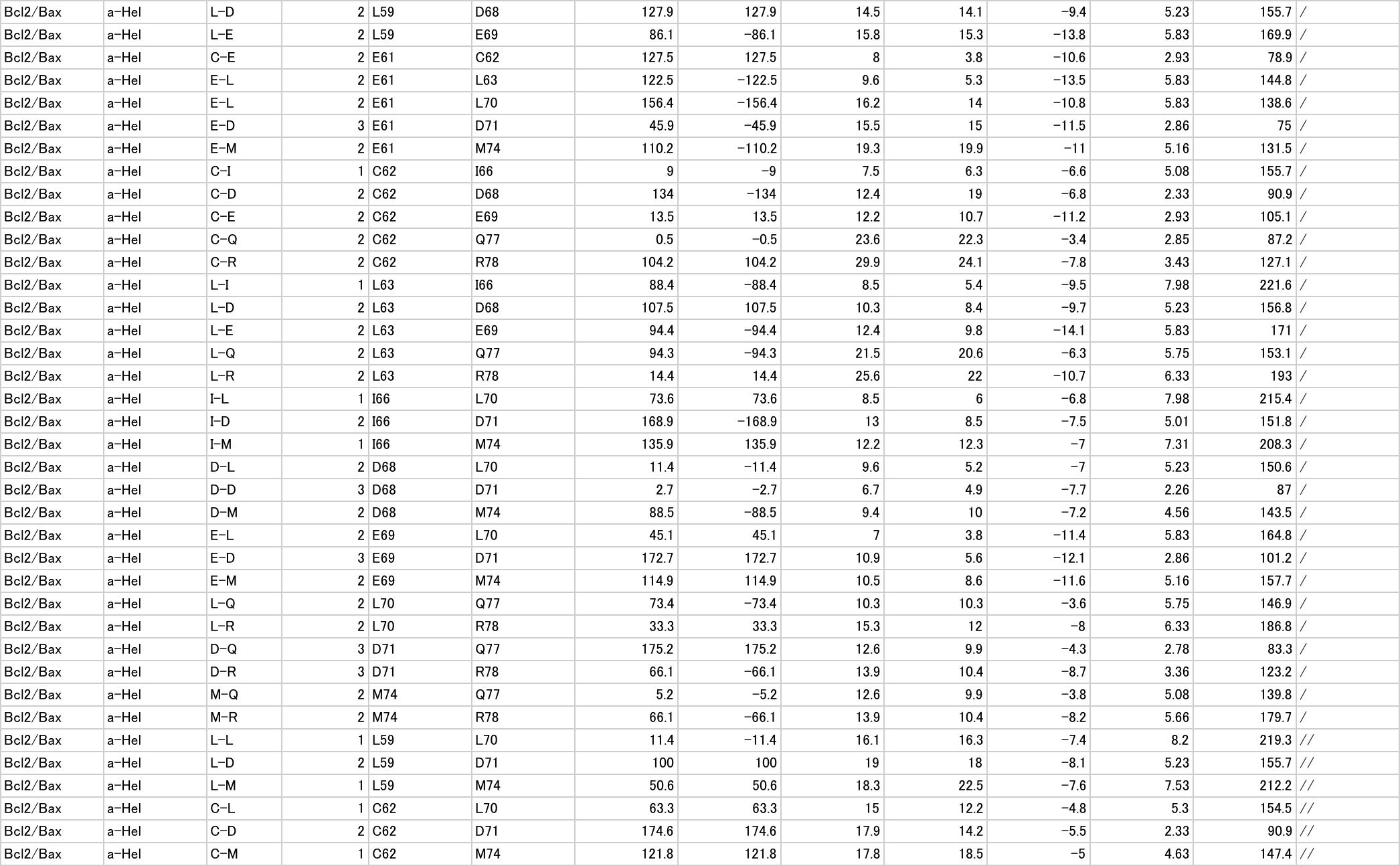
**

**Supplementary Table 9: All structural data**


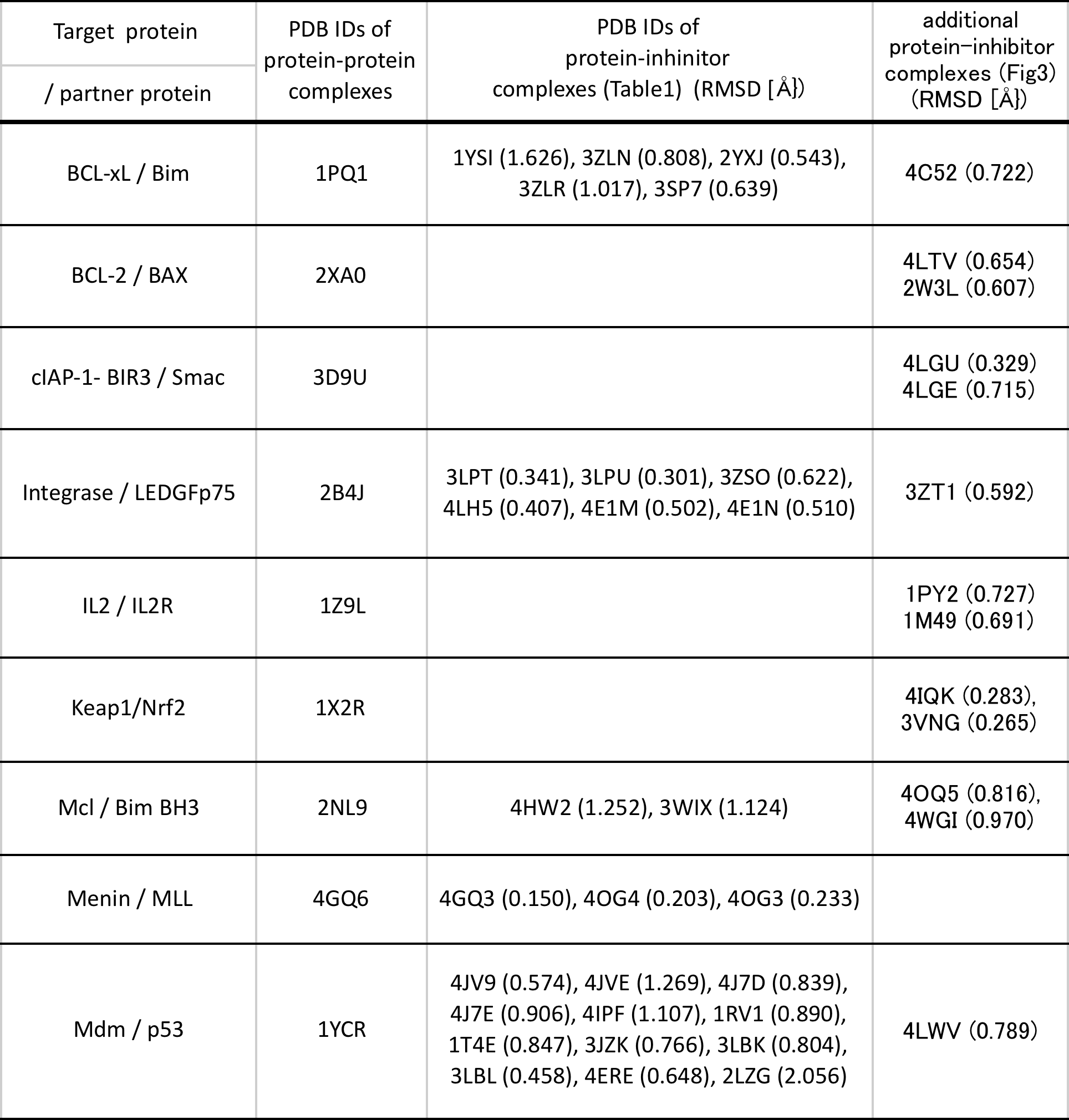


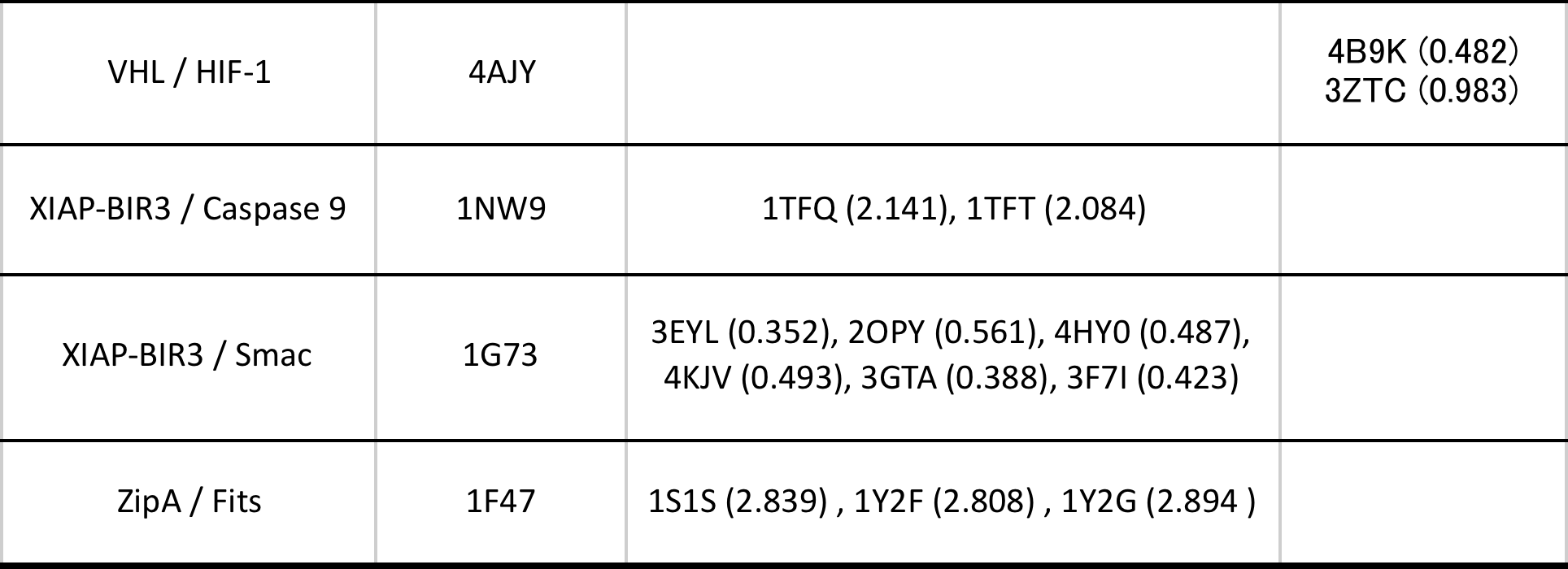


^a^ Each RMSD was calculated by Pymol.

**Supplementary**
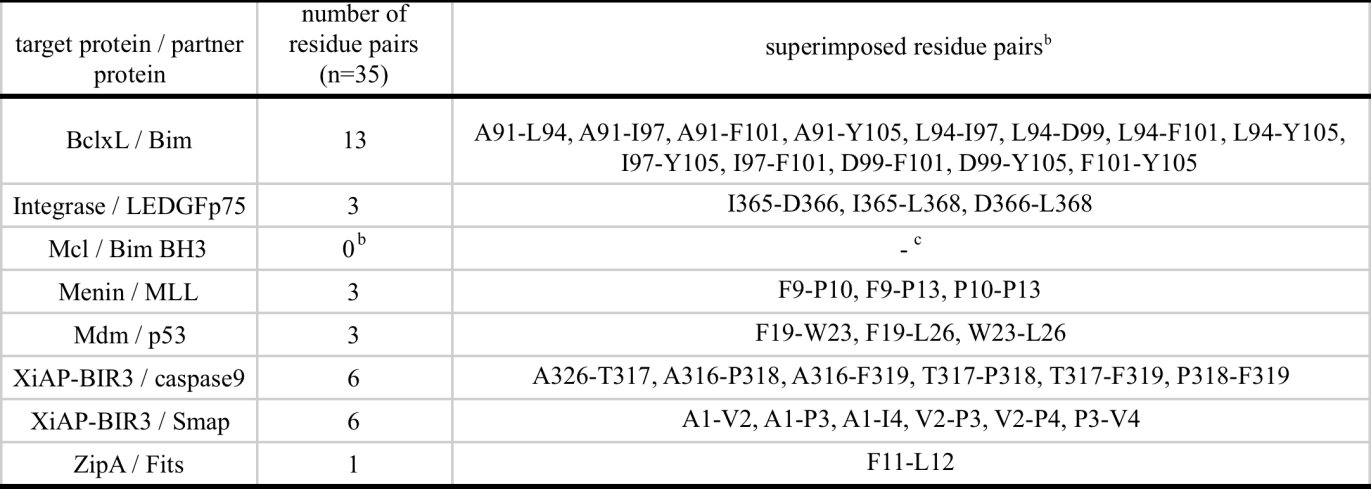
**Table 10: Residue pairs (n=35) that were superimposed onto 39 inhibitors**

^a^ Every combination of superimposed residues on Table 1 was not listed on this table because superimposition of some residue pairs with 39 inhibitors was not observed.

^b^ Pairs of residues that were superimposed on small molecules.

^c^ The residue (L62) was only superimposed onto small molecules (PDB: 4HW2 and 3WIX), so there was no pair.


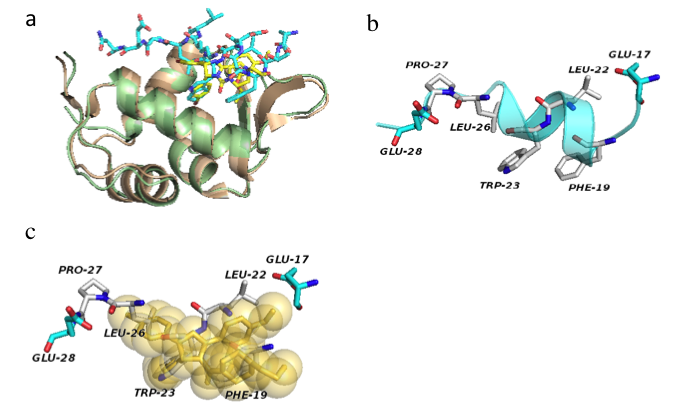


**Supplementary Figure 1:** An example of how the superimposition of residue pairs with an inhibitor can be defined. The structure of Mdm-p53 was used as an example. The target protein was Mdm and the partner protein was p53. The figures were produced using Pymol. (a) Computational alignment. The alignment between Mdm of the protein–protein complex (pdb:1YCR, light green) and Mdm of the small molecule–protein complex (pdb:1RV1, wheat) are shown. The p53 peptide (cyan) and the inhibitor (yellow) are shown. (b) An example of selected residues from the ANCHOR db (pdb:1YCR). (c) An example of the superimposition of residues with a small molecule. In this case, three residues (F19, W23, and L26) and three residue pairs (F-19-W23, F19-L26, and W23-L26) were defined as being superimposed onto the small molecule (pdb:1YCR).


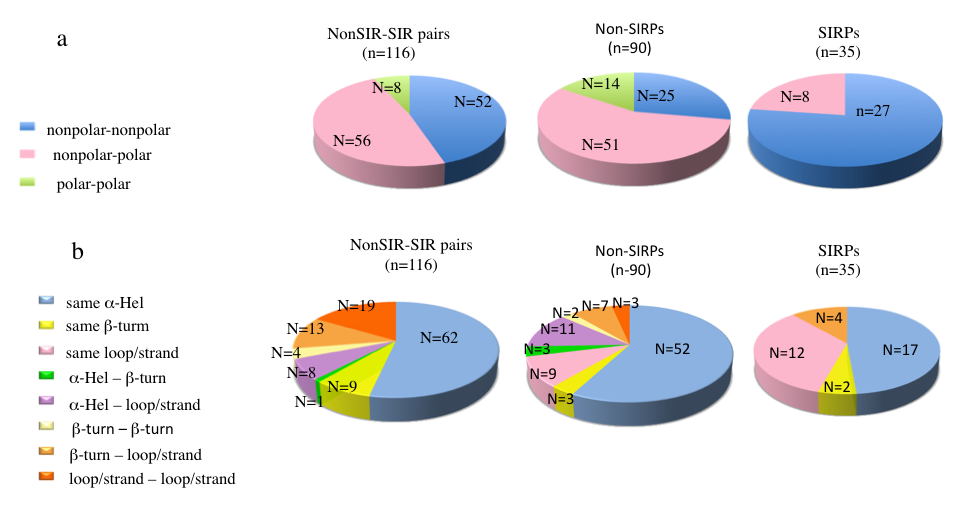


**Supplementary Figure 2:** **The classification of the residue pairs that were not superimposed on inhibitors (nonSIRPs, n=90), and residue pairs that one was superimposed with an inhibitor and another was not (nonSIR-SIR, n=116), residue pairs that were superimposed on inhibitors (superimposed residue pairs, n=35)**  (a) Classification of residue pairs on the basis of polarity of amino acids’ side chains. Residue pairs were classified into 3 groups (nonpolar–nonpolar residue pairs (blue), nonpolar–polar residue pairs (pink), polar–polar residue pairs (green)). (b) Classification of secondary structure combinations. Both residue pairs were classified into 8 groups (two residues on the same α-Helix (light blue), on the same β-turn (yellow), on the same loop/strand (pink), one on the α-Helix and the other on the β-turn (α-Hel–β-turn) (light green), α-Hel–loop/strand (violet), β-turn–β-turn (light yellow), β-turn–loop/strand (orange), loop/strand–loop/strand (red)). There was no α-Helix–α-Helix structural combination of residue pairs in 243 pairs.


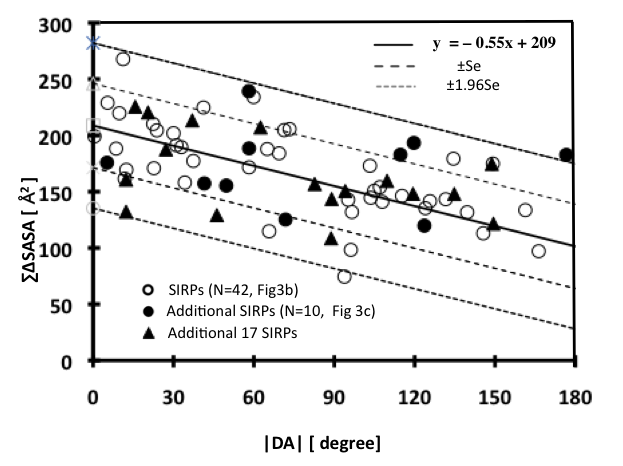


**Supplementary Figure 3: 17 additional SIRPs spotted on Figure 3c**
